# Attitudes of patients with anorexia nervosa to compulsory treatment and coercion

**DOI:** 10.1016/j.ijlp.2009.10.003

**Published:** 2010-01

**Authors:** Jacinta O.A. Tan, Anne Stewart, Raymond Fitzpatrick, Tony Hope

**Affiliations:** a The Ethox Centre, Department of Public Health and Primary Care, University of Oxford, Oxford, United Kingdom; b Oxfordshire and Buckinghamshire Mental Health Partnership Foundation NHS Trust, United Kingdom; c Department of Public Health, University of Oxford, Oxford, United Kingdom

**Keywords:** Anorexia nervosa, Compulsory treatment, Coercion, Patients' views, Qualitative interview study

## Abstract

**Background:**

The compulsory treatment of anorexia nervosa is a contentious issue. Research suggests that patients are often subject to compulsion and coercion even without formal compulsory treatment orders. Research also suggests that patients suffering from anorexia nervosa can change their minds in retrospect about compulsion.

**Methods:**

Qualitative interviewing methods were used to explore the views of 29 young women concerning compulsion and coercion in the treatment of anorexia nervosa. The participants were aged between 15 to 26 years old, and were suffering or had recently suffered from anorexia nervosa at the time of interview.

**Results:**

Compulsion and formal compulsory treatment of anorexia nervosa were considered appropriate where the condition was life-threatening. The perception of coercion was moderated by relationships. What mattered most to participants was not whether they had experienced restriction of freedom or choice, but the nature of their relationships with parents and mental health professionals.

**Conclusions:**

People with anorexia nervosa appear to agree with the necessity of compulsory treatment in order to save life. The perception of coercion is complex and not necessarily related to the degree of restriction of freedom.

## 1 Background

Anorexia nervosa is a mental disorder which often leads to serious risk of physical harm or even death to the individual, through self-imposed dietary or other behavioural strategies aimed at losing weight and self harm (Harris & Barraclough, 1997, 1998). However, there is controversy over whether compulsory treatment for anorexia nervosa is appropriate (Draper, 2000; Giordano, 2003; Tiller, Schmidt, & Treasure, 1993).

Compulsion is not solely achieved through legal measures. Some mental health professionals use not only formal legal powers to compel patients to have treatment, but also the threat of legal orders or other powers as ‘leverage’ to obtain agreement to treatment (Appelbaum & Redlich, 2006; Carney, Tait, Richardson, & Touyz, 2008; Carney, Wakefield, Tait, & Touyz, 2006). In cases where patients are legal minors, it is common to use other means of compulsion such as parental consent (Ayton, Keen, & Lask, 2009). Psychiatric patients' perceptions of coercion are complex and not directly correlated with the use of compulsory legal orders (Bindman et al., 2005; Rajkumar, Saravanan, & Jacob, 2006; Salize & Dressing, 2005; Watson, Bowers, & Andersen, 2000). One study found that patients with anorexia nervosa experience high levels of ‘perceived coercion’—that is, the perception that they are being coerced whether or not formal mechanisms are used. Some of these patients changed their views in hindsight about the coercion that they had received (Guarda et al., 2007). Most research in this area has focussed on using quantitative measures of perception of coercion. There have been few in-depth studies exploring the views of patients who suffer or have suffered from anorexia nervosa, about their experiences of coercion and compulsory treatment.

## 2 Method

A qualitative interview study was carried out to determine the views of people with anorexia nervosa as well as their parents with respect to compulsory treatment, treatment decision-making and competence. In this article we report the patient participants' views concerning compulsory treatment.

The qualitative interviews were semi-structured and the interview was conducted using a topic guide, which served as a springboard for wider, more flexible and unstructured narratives and discussions. Participants were asked to talk about their own experiences of treatment, and the interviewer encouraged elaboration of experiences relating to the three main foci of the study given above. Questions in the topic guide relating specifically to compulsory treatment are listed in Box 1. As will be seen the questions ask about whether it is acceptable to ‘make people have treatment’ or ‘to be treated even if they don't agree’. The concept of coercion and compulsory treatment was thus left to participants to interpret as they saw fit and was not defined in terms of legal processes.

Box 1 **Excerpt from topic guide for patient participants.**

*Attitudes to use of compulsion*

1. *Attitudes to compulsory treatment of any kind* - Do you think it’s ever acceptable to make people have treatment when they don’t want it or agree to it?
2. *Attitudes to compulsory treatment of mental disorders in general* - Do you think people with mental illnesses like schizophrenia should, under some circumstances, be treated even if they don’t agree with the treatment? Why?
3. *Attitudes to compulsory treatment of anorexia nervosa in particular* - Do you think there are some circumstances under which people with anorexia nervosa should be treated even if they don’t agree with the treatment? If so, what are these circumstances?
• *when is it justified?*
• *when is it effective?*

The interviews were audio-taped and transcribed, with names and places removed. The coding framework categorising the broad issues discussed in the interviews was developed through repeated readings of the transcripts, followed by trials of application to transcripts and discussions between two coders who coded some transcripts independently. Each transcript was then coded using the final coding framework. Common emergent themes as well as divergent themes within each category of the frame were further analysed. The N6 qualitative software programme was used to assist the coding process and collation of themes and subthemes (QSR International, 2005).

## 3 Participants

Twenty-nine patients with current or recent anorexia nervosa were recruited from four different treatment centres in southern England, which covered a range of characteristics: private and National Health Service, adolescent and adult treatment services, specialist eating disorder centres and general mental health units. The patients' ages ranged from 15 years 10 months to 26 years 2 months (median 17 years 0 months, mean 18 years 1 month). Note that in England the age of legal majority is 18 years, which is also usually the age at which patients move from adolescent to adult mental health services. Although one of the treatment centres did allow goals of ‘maintenance’ for certain adult patients not yet ready to accept treatment, for all the participants in this study the treatment from the mental health services consisted of a combination of a weight restoration as well as psychological therapies. Agreement to treatment therefore meant accepting both weight restoration and psychological treatment.

Patient records were not accessed, so all information was obtained from the participants themselves. Participants' self-reported Body Mass Indices (BMI) ranged from 12.4 (dangerously underweight) to 28.4 (overweight, technically ‘pre-obese’), with a mean body mass index of 17.7 (below normal range) and a median BMI of 17.65. By their own accounts, the participants were at various stages of illness, treatment and recovery at the time of interview.

Of the 29 participants, eight were inpatients in mental health units although at the time of interview none was detained under the Mental Health Act 1983.^1^ Eighteen participants were either day patients or outpatients utilising mental health services at the time of interview. One participant was waiting to have treatment. One participant had been discharged by the eating disorder service after declining an offer of inpatient treatment for low weight. One participant had chosen not to accept treatment as she had a previous aversive experience of inpatient treatment.

Of the eight inpatient participants, five described themselves as having been admitted without free choice, either owing to parental pressure or under the implied or overt threat from the mental health professionals of a Mental Health Act 1983 ‘section’ (compulsory detention order for the purposes of assessment or treatment) if they did not comply. Two of these had subsequently been placed on a Mental Health Act 1983 Section 3 (a compulsory detention order for treatment of a mental disorder) during the course of their admission and both had been recently discharged from the Section 3 order at the time of interview. One of these two patients had also experienced being detained using the Mental Health Act 1983 during a previous admission. Only three of the eight inpatients, therefore, described themselves as having made a free choice to be admitted to hospital for the current admission, and one of these three participants described a previous inpatient admission to a different unit to which she had not given consent.

Only three of the 18 day patient and outpatient participants said they had made a choice to enter and remain in treatment on their own. Six participants described being coerced into having treatment against their will. A further nine described either shared decisions concerning treatment made together with doctors and parents (with varying degrees of pressure from professionals and parents ranging from encouragement to ultimatums), or decisions about treatment made by doctors and relatives on their behalves with their tacit agreement. It is important to note that the Mental Health Act 1983 did not enable compulsory outpatient or day patient treatment, so no formal compulsory treatment under mental health legislation would have been possible.

Only two out of the 29 participants (6.9%) had ever experienced formal compulsory treatment, both under the Mental Health Act 1983; but 15 of the 29 participants (51.7%) gave accounts of having experienced loss of freedom of choice regarding treatment either during their current treatment or in the past. Types of loss of freedom of choice included ‘leverage’ in the form of threats (overt or implied) of compulsory admission, other types of compulsion such as parental consent for treatment, or restriction of choices such as only being allowed to choose between types of treatment (for example, inpatient or outpatient) but not whether to have treatment. These figures are consistent with the published literature. This literature shows that a relatively low proportion of inpatients with anorexia nervosa are placed on formal compulsory treatment orders, with reports ranging from 9% to 28% (Carney et al., 2008; Ramsay, Ward, Treasure, & Russell, 1999; Royal College of Psychiatrists, 1992; Watson et al., 2000). A user survey, however, suggests that a much higher proportion of patients perceive a lack of choice regarding treatment (Newton, Robinson, & Hartley, 1993), and some studies suggest that ‘leverage’ is commonly used by psychiatrists with a significant minority of psychiatric patients in order to increase compliance with treatment without resorting to legal compulsion (Appelbaum & Redlich, 2006; Bindman et al., 2005).

## 4 Results

### 4.1 Experiences of compulsion in ‘voluntary’ treatment

In this article, we will use the term ‘compulsion’ to indicate a restriction or removal of free choice with regard to having treatment; ‘formal compulsion’ to indicate compulsion using legal treatment orders; and ‘coercion’ to indicate a negative perception of a loss of choice or freedom. Participants described many pressures to accept ‘voluntary’ treatment without the use of legal powers and experienced these as restricting their freedom of choice with regard to treatment. Box 2 gives four examples.

Box 2 **Experiences of compulsion in ‘voluntary’ treatment.**

**My dad was very heavily involved and V and Dr. P have been involved in it quite a lot, (3 seconds) but (4 seconds) I mean I came, technically I came in here voluntarily, and technically I suppose it was my decision …but it doesn’t always feel like that when there’s a lot of pressure and a lot of guilt, that’s played a big part in it. And personally I was just left feeling that ….there really was no other choice. 39P**

**I was meant to go as an inpatient at the P [adolescent general psychiatric unit] about two years ago, but I didn’t want to, and so I did the treatment at home. And I didn’t really think I had a choice [about whether to have treatment], looking back on it now I think: “Well, why didn’t I just not eat?” And I didn’t want to, but I still did it because I thought I didn’t have a choice. So in a way that was sort of forced upon me, I didn’t want to get better then. 19P**

**Well, I was given two options really. Either I refused, and they said “if you refuse to put on weight we’re taking you straight up to the hospital and you’re on tubes and drips and everything and, you know, you’re going to have to gain weight or, and you’ll be made an inpatient.” So they said they’d put me in a hospital first to get me up [in weight], just on kind of whatever and then I’d become an in-patient. And, *or* I was an outpatient but I gained weight. So I chose the outpatient version because if I went, if I had to become an inpatient I would be in there for five or six months, they told me, with the amount of weight gain I had to have. And I wouldn’t want to be in a mental institution for six months, that sounds kind of incredibly depressing to me. 16P**

**I was desperate, I didn’t want to do it, I did want a way out but I also didn’t, if you know what I mean. It comforted, it made me feel *disgusting* but it comforted me in a way, and so I didn’t want to give it up and the only reason that I came to X [adolescent treatment centre] was because they said that if I didn’t come voluntarily I’d be Sectioned and I didn’t want that on my record. 12P**

### 4.2 Attitudes to the use of formal compulsion for mental disorders

All participants thought that formal compulsory treatment under the Mental Health Act 1983 was justified for some individuals. Indeed, participants thought that mentally disordered patients, for example those with schizophrenia, have a right to be treated, compulsorily if necessary, in order to protect themselves and others from harm. Participants viewed such compulsion as supportive and helpful. Many participants justified such compulsory treatment in terms of the effect of mental disorders in limiting the ability to understand and decide on the need to have treatment (see Box 3).

Box 3 **Attitudes to the use of formal compulsory treatment in mental disorders.**

**I don’t know much about schizophrenia, but like I said, if they’re a danger to themselves or other people or even sometimes if they’re living alone. Then yeah, I’d say they need support, they need help. 18P**

**I suppose in a way, yes, if it’s going to cause harm to someone else. So schizophrenia, if they’re causing, yeah if they’re going to cause harm to other people then yes, I think it’s right that you know like: murder, death, which you do hear about, so at that stage yes I think we should be [treating them against their will], and I suppose if they are really at risk of death to themselves. 26P**

**I suppose a *mental* disorder though, the thing is they might have a mental problem which is screwing up their view of whether they need it or not, they have it, like in anorexia. So physically they do need it. 20P**

**I think that when one is in the hands of mental illness and there are times when you’re aware you’re thinking in ways which really aren’t natural to you, there are moments, I think, of lucidity when you crave for help and I don’t see that a schizophrenic is any less needy [of help] in those terms. 24P**

### 4.3 Attitudes to the use of compulsion in anorexia nervosa in order to save life

With respect to anorexia nervosa, the participants all agreed that overriding treatment refusal using compulsion, including formal compulsory treatment (e.g. under the Mental Health Act 1983) if required, was necessary in order to save life (see Box 4). This was generally seen as self-evidently the right thing to do because no one should be allowed to die from the consequences of having anorexia nervosa. This view was often based on personal experiences of compulsion and risk to self. Many participants spoke about how they had resisted or disagreed with treatment under compulsion at the time, but were grateful for it in hindsight.

Box 4 **Attitudes to the use of compulsion in anorexia nervosa.**

Justification for formal compulsory treatment is to save life

**I meant the taking charge, is when basically when someone’s at the point where they just collapse and you need to put them on drips and that sort taking charge. But I mean I don’t think with anorexia there’s going to be any other point where you really need to completely take charge with them. 16P**

**I think if somebody’s life is in danger and is threatened and they have to go into hospital then yes it’s very important to obviously re-feed them and to get them to a stage where they’re not, where they’re medically stable, but you can’t enter anybody into treatment if they’re not willing to. 36P**

Approval of the use of compulsion (with gratitude in hindsight)

**I think other people should be made to have treatment because you do get to the point where you don’t know what’s right for you. I know last year when I was ill there was no way I would have let anybody do anything to treat me, like for my own choice I would have just carried on losing weight, I know I would have done until I didn’t live anymore, but now I hate to think that just because I said “no” I would have been left. 30P**

**If I had been left without somebody forcing treatment upon me I would have just starved myself to death. So, you know, I wouldn’t have got to my target weight and got happy and have things that I’ll have in the future. 21P**

**So then although when I was back there [i.e., very ill] I’d say “no, that’s a stupid idea,” now being *here* I look back on it, I think “hell yeah, you can’t *not* treat someone who’s going to die because they’re starving themselves.” 20P**

Need for compulsion because of inability to make decisions

**I suppose if you just let people carry on losing weight then they can die of a heart attack, and they might in the future have wanted to get better; so you’ve got to, if people are in danger, you’ve got to get them to that stage where they want to get better. 19P**

**I think ultimately beating anorexia has to be a decision that you make yourself. But if your health is so bad that you’re dying or you’re at risk of very, very severe illness, then I think you should be treated until you can make the decision. Because I think if you’re ill enough you can’t make that kind of decision. 22P**

Perceptions of Mental Health Act 1983 treatment orders

**I don’t really know what a [Mental Health Act 1983] Section 3 entails. I mean for me I thought it was, well for one, it makes you look mental. And it doesn’t look good when you’re looking for a job, on your CV, and its written down, you were on Section 3 or I’ve had you know and it plays an effect on going abroad I think, someone said to me. So I don’t really know a lot about it but I mean if that’s what’s going to happen for this poor girl or, or boy of, you know 11, 12, 13, 14, 15, 16, 17, that’s really horrible. They don’t want that put on their lives at such a young age. So if that entails the parents [use of parental consent] I would definitely go with the parents’ point of view. 18P**

Some participants considered that informal compulsion from professionals and families was also acceptable to save life. The central reason that participants gave to justify compulsory treatment was that no one should die from a treatable disorder as such a death would be a waste of life. Several participants also argued that the acceptance of a high risk of death was not usually the true or fixed view of people with anorexia nervosa but rather a result of the disorder. Furthermore, at the extremely low weights and poor physical condition where people are at risk of death, participants felt that patients suffering from anorexia nervosa are unable to make their own decisions. Participants saw saving patients' lives as having the effect of saving them to think about accepting treatment another day. In these dire circumstances, the views of patients were thought to be suspect or even irrelevant, as the disorder would be dominant and would be driving the wishes and behaviour of the person with respect to treatment.

Participants did not universally view ‘leverage’ and other non-legal forms of restriction of choice as negative or unethical; indeed, formal compulsion through legal means was generally seen as a worse option than these more informal means of compulsion. There was a view that being placed on a compulsory detention order would be highly stigmatizing, and would affect their future, particularly with regard to their careers (see last quotes in Boxes 2 and 4). Some participants also had a perception of greater restriction with formal compulsory treatment orders, mainly through the perceived losses of personal freedom and the ability to negotiate terms of their treatment.

### 4.4 Attitudes to the use of compulsion in anorexia nervosa in order to treat the disorder

In contrast with the universally held view that formal compulsion through legal procedures was justified to save life, there was a spectrum of views concerning the use of compulsion to treat the disorder itself (see Box 4). Two participants felt that formal compulsory treatment could be useful in an early stage because the disorder would be relatively easy to treat and timely treatment could prevent a great deal of future suffering. The general view, however, was that formal compulsory treatment in the absence of life-threatening illness was neither desirable nor helpful in terms of achieving true recovery. The arguments against the use of compulsory treatment were both its inefficacy and unfeasibility. Most participants thought that going through treatment and achieving recovery required some degree of consent and cooperation from the person herself. Indeed, there was a commonly held view that the inappropriate use of compulsion could be harmful in itself.

Participants' views about the use of informal means of compulsion, coercion and pressure to comply with treatment were more complex. Many participants held the view that people with anorexia can become less able to make treatment decisions because of four distinct problems: a sense of anorexia nervosa being a part of their personal identity, the issue of control and loss of control, changes in values due to the disorder itself, and difficulties in thinking about the risks involved. This result validates the findings of a previous smaller study reported elsewhere (Tan, Hope, & Stewart, 2003a,b; Tan, Hope, Stewart, & Fitzpatrick, 2003c; Tan, Hope, Stewart, & Fitzpatrick, 2006). These issues provide grounds for doctors and nurses to use some pressure or restriction of choice in order to deal with refusal or reluctance to engage with treatment. Participants also described how the context and relationships within which treatment decisions were made were crucial to the participants' perceptions of compulsion or choice. This is taken up in the next section.

### 4.5 The context of treatment decisions and how they affect the experience of compulsion

A major emergent set of themes concerned context and relationships. Having freedom of choice was often less important to participants than their relationships with, and the attitudes of, those around them and treating them. Many described decisions made on the basis of trust and good relationships rather than on the basis of the elements highlighted by most theoretical, clinical and legal descriptions of capacity such as understanding treatment information, retaining it, reasoning and weighing it up, and communicating a choice (Grisso & Appelbaum, 1998; Roth, Meisel, & Lidz, 1977).^2^

Some participants who had not been given free choice did not appear to resent their experiences nor perceive them as coercive. Instead, they viewed the strong influence, pressure, supervision or restrictions imposed by parents and professionals as helpful, caring and supportive (see Box 5). In some cases, this was to the extent that they felt it would be inappropriate for parents or professionals to place the sole responsibility of making a decision on the patient herself.

Box 5 **Experience of the restrictions of choice in treatment as helpful and supportive.**

**I think it [the treatment] is really good, really appropriate for me, anyway. I think they’re quite good at getting the balance between giving you responsibility and freedom without giving you too much. (Laughs) Giving you more than you can cope with. Like they don’t sort of, the freedom they give you doesn’t jeopardise your recovery, it sort of makes it more bearable. 19P**

**Do, you need help, you need treatment, you need to come into a place where you can get extra support, extra external help, you need to hand over the controls because you’re not in control anymore, it deludes you in thinking you are in control but you have to, that’s where you have to stand back or somebody else has to stand back for you and say look you are not in control, it is completely controlling you. 36P**

Some participants did resent the supervision and restrictions imposed on them and viewed these restrictions as having been unhelpful in the process of recovery (see Box 6). For these participants the key reason for this resentment and sense of coercion was the feeling that they had been dismissed, belittled, or punitively treated in the process of receiving treatment rather than the nature and extent of the restrictions themselves. These participants described feelings of being stripped of their individuality and having had their wishes ignored within overly restrictive regimes which felt inappropriate to their requirements for consultation or personal choice.

Box 6 **Experience of the restrictions of choice in treatment as unhelpful and coercive.**

**If I refuse some food because I just don’t want it, everyone’s thinking “oh she’s not having that, why is she not having that, she should be having that!” and then the feeling of being watched over; and [I would like them instead to be] kind of letting me get on with it myself, but knowing that I could go and talk to them and it wouldn’t affect them, they wouldn’t suddenly run round and say “have this, do this, do that!” but at the same time it’s being able to speak to someone and to say to me “yes, you know you can”, that’s good. 26P**

**And also that’s something I find it very hard to talk about, it’s very hard to just say [to the psychiatrist] you know “well actually, you know, you’ve pushed me too far and I’m now throwing up after every meal”. I mean you can’t really say that to someone, it’s very hard to say that, you know. And also I reckon that obviously if I did say that, I just didn’t want to just have to think about the restrictions that would then be placed on me. I’d just be even more restricted. But you know I wouldn’t have been pushed to that [i.e., becoming bulimic] if I’d been given more choice about [how slowly I gained weight] perhaps you know, because I was gaining weight, it wasn’t as if there was an issue with me gaining weight, I was just doing it more slowly. And I was doing it in a way that I was comfortable with. 16P**

The participants' relationships with parents and professionals emerged as an important factor in decision-making. To some participants, the issue central to the decision was not whether treatment was perceived by them as being the best course, but whether they were able to trust their parents and professionals enough to overcome their own ambivalence and reservations. It was also their relationships with these people that helped them to decide to engage with and accept treatment, which was often restrictive in terms of diet plans, regimentation of their lives and strict supervision (see Box 7). Two participants stated how they decided to have treatment not because they wished to recover, but because having anorexia nervosa was causing their parents distress.

Box 7 **Trust and relationships and their impact on acceptance of restrictions of choice.**

**I mean trust was definitely the *key* issue with me. Because if you don’t trust your doctors or you don’t trust your parents to be doing what they think is best, then you’re not going to do it. So yeah, I think that I had good doctors, because they really understood. And they all, it was always sort of, from my, it didn’t feel like they were all ganging against me, it felt like they were coming from my point of view. Which made it much easier to trust them. And, yeah, I feel, I feel that it’s been done well. 14P**

**I think that (3 seconds) again from just a very personal point of view I know that there was a time when I *couldn’t* eat enough to make me live or whatever and therefore I had, I was put in a position where I still accepted although I resisted, but where I was told I had to, black or white. I think physically you reach a stage where yes, you need that, psychologically it can be quite damaging. But I do think that in the most extreme initial phases, that yes, maybe it [i.e., use of compulsion] is necessary but always, *always* listening and even when a patient resists to listen to why they are resisting, not to assume you know why they are resisting. Because then that’s how you best help them. 24P**

Participants described how a poor relationship with either parents or professionals resulted more from punitive, dismissive or disrespectful behaviour and failure to listen carefully and respectfully to their feelings and wishes, than from compulsion or the restriction of choice itself (see Box 6). However, several voiced the opinion that it was better for compulsion to be exerted by professionals than by parents, as parental coercion could result in resentment and damaged personal relationships. Compulsion from professionals, by contrast, was perceived as part of the professionals' job even when participants did not agree with it.

## 5 Discussion

Whatever their views about the use of compulsion in anorexia nervosa in general, or on issues of competence and capacity, all participants thought that it is right to impose treatment in order to save life. No participant supported the view that it is right to allow a person with anorexia nervosa to die as a result of respecting their refusal of treatment.

A concept that is receiving increasing research attention is that of ‘perceived coercion’ (Bindman et al., 2005; Guarda et al., 2007). This is the idea that psychiatric patients often experience coercion whether or not formal legal means are used. Such coercion includes ‘leverage’ when a patient may be ‘encouraged’ to behave in a certain way through a threat of the use of more formal coercion or the loss of some benefit (Appelbaum & Redlich, 2006). The participants in this study described such perceived coercion. Indeed their experience of formal legal compulsion was much less frequent than that of other forms of compulsion.

The participants' frequent accounts of experiences of lack of choice and the use of leverage are ethically problematic when contrasted with the relative infrequency of legal compulsion they experienced. On the one hand, the use of leverage can be an effective and flexible way of enabling acceptance of treatment while avoiding the stigmatizing and potentially traumatic experience of formal legal compulsion, and may not be experienced negatively; on the other hand, the use of leverage and other non-legal forms of coercion leave already vulnerable patients at risk of unethical treatment and loss of autonomy and rights without the protection of legal procedure, advocacy and right of appeal that formal legal compulsion brings. It has been argued that it is unethical and discriminatory towards those with mental disorders to use any form of coercion or act in a patient's best interests without consent unless he or she clearly lacks capacity (Szmukler, 2001); and furthermore, that coercion is counterproductive in anorexia nervosa (Rathner, 1998). Studies suggest that patient perception of ‘coercion’ or experience of formal legal compulsion is not subsequently associated with a poorer engagement with treatment or poorer therapeutic relationship with mental health professionals (Ayton et al., 2009; Bindman et al., 2005; Greenberg, Mazar, Brom, & Barer, 2005). The limited research also does not clearly show that formal compulsory treatment is associated with poorer short or longer term clinical outcome (Ayton et al., 2009; Carney et al., 2008; Ramsay et al., 1999; Watson et al., 2000). Even if there is no clinical harm, however, the issue of compulsion in treatment for anorexia nervosa is still ethically problematic. From the ethical point of view, a traditional stance is that patient autonomy should be respected even if unwise or foolish decisions are made, and the justification for acting in a patient's best interests instead of according to his or her wishes is the pivotal issue of capacity (Buchanan & Brock, 1989).

What is striking from this study is that the issue of capacity was not the central one for participants in the context of compulsory treatment. It is true that many participants did view treatment refusal by people with anorexia nervosa as problematic because the anorexia compromises decision-making. But this was not the main issue in their consideration of the rights and wrongs of compulsion. The main issues centred on their relationships with those involved in the treatment: health professionals and sometimes parents. The participants did not appear to resent leverage or ‘informal’ compulsion if it is carried out in the context of a trusting relationship and perceived as care and help, and may not even experience it as coercive.

These results have implications for the ethical analysis of compulsory treatment in the context of anorexia. One approach, we suggest, is that, on the accounts of those with anorexia, the central issue is not the question of whether and when a person lacks capacity to consent to, or refuse, treatment. Instead the central issue concerns the context and relationships involved in good and compassionate psychiatric care. A greater objective restriction of choice may be experienced as less negative, and indeed ultimately as good care, if conducted within a trusting and supportive relationship and environment. Such an approach might be seen as a ‘virtue ethics approach’ in which the focus is on how carers can act with compassion. It is also consistent with recent trends in bioethics that highlight the importance of trust in decision-making (O'Neill, 2002).

An alternative response to the accounts reported here is to retain the emphasis on autonomy and capacity in thinking about the ethics of compulsion but to suggest that more subtle and broader difficulties in making treatment decisions may need to be considered (Charland, 1998). Key questions are: when and why a patient may fail to make autonomous decisions because of the influence of the mental disorder, and whether help and even coercion to engage in treatment might increase autonomy (Charland, 2002; Tan & Hope, 2006; Tan et al., 2006). Conversely, a patient may technically lack capacity, for example being impaired in his or her ability to retain information, but broader factors such as an ability to express underlying values may enable him or her to continue to make valid choices with the appropriate support (Department of Constitutional Affairs, 2007; Tan et al., 2006).^3^

These two approaches are not mutually exclusive, as poor relationships and difficult or hostile settings tend to disempower patients with respect to whether they feel able to make decisions for themselves and can also cause them to resist or refuse help; while strong, trusting relationships and supportive treatment settings tend to facilitate patients in making their own decisions and help them to feel more actively involved in their care. Indeed, the ethical justifications voiced in these results suggest that it is a complex mix of a virtue ethics and nuanced capacity approach that is used by patients in thinking of their experiences of, and attitudes to, compulsory treatment and coercion. The analysis of this study and results of a previous study suggest that in anorexia nervosa issues of control and choice can have a strong influence on whether patients are able to make decisions. This is because patients suffering from anorexia nervosa often struggle to remain in control of themselves and their lives and, as a result, resist help, while at the same time feeling out of control with respect to their eating behaviour and wishing to have help (Tan et al., 2003c).

In conclusion, in this qualitative interview study, patients with anorexia nervosa reported considerable experience of compulsion and restriction of choice despite a relative lack of the use of formal compulsory treatment. Nevertheless, there is strong consensus that compulsory treatment should be used if needed to save life in anorexia nervosa. There was less consensus concerning the use of compulsory measures for the treatment of anorexia nervosa in the absence of immediate risk to life. A striking result was that what mattered most to participants was not whether they were compelled to have treatment but the nature of their relationships with parents and mental health professionals. Indeed, within a trusting relationship compulsion may be experienced as care.

## Footnotes

1 Note that the research interviews were conducted from 2002 to 2006, before the Mental Health Act 2007 amendment to the Mental Health Act 1983 became law.

2 In England and Wales the relevant legislation is The Mental Capacity Act 2005, which has the following definition of (in)capacity: *“*a person is unable to make a decision for himself if he is unable (a) to understand the information relevant to the decision, (b) to retain that information, (c) to use or weigh that information as part of the process of making the decision, or (d) to communicate his decision (whether by talking, using sign language or any other means).*”*

3 The Mental Capacity Act 2005: Part 1, 1: The Principles (3) A person is not to be treated as unable to make a decision unless all practicable steps to help him to do so have been taken without success.
